# Cell death of spinal cord ED1^+^ cells in a rat model of multiple sclerosis

**DOI:** 10.7717/peerj.1189

**Published:** 2015-08-13

**Authors:** Dragana Trifunović, Neda Djedović, Irena Lavrnja, Katrin Sophie Wendrich, François Paquet-Durand, Djordje Miljković

**Affiliations:** 1 Institute for Ophthalmic Research, University of Tuebingen, Tuebingen, Germany; 2 Department of Immunology, Institute for Biological Research “Siniša Stanković”, University of Belgrade, Belgrade, Serbia; 3 Department of Neurobiology, Institute for Biological Research “Siniša Stanković”, University of Belgrade, Belgrade, Serbia

**Keywords:** ED1, Cell death, Gray matter, Caspase 3, Macrophage, Microglia, EAE

## Abstract

Infiltration of macrophages into the central nervous system and activation of microglia are hallmarks of multiple sclerosis and its animal model—experimental autoimmune encephalomyelitis (EAE). Cell death in EAE has been demonstrated as an essential mechanism in the local regulation of the inflammatory reaction, but also as one of the major factors contributing to the destruction of the nervous tissue. The focus of this study was on detection of cell death among ED1^+^ cells (macrophages/activated microglia) in the spinal cord of Dark Agouti rats at the peak of EAE. Cell death was assessed using the TUNEL assay and immunostaining for cleaved caspase 3, as markers for cell death in general and “classical” apoptosis, respectively. Major infiltrates of immune cells were detected both in white matter and gray matter of spinal cords in rats at the disease peak. ED1, TUNEL, and caspase 3 positive cells were detected within, but also outside the infiltrates. There were more dying ED1^+^ cells in white matter than in gray matter, both in the general population and in infiltrated regions. The observed discrepancy in the proportion of dying ED1^+^ cells in spinal cord gray and white matter indicated that in EAE rat macrophages/microglia within gray matter are less prone to cell death induction. This is of special interest in the context of the increasingly appreciated contribution of spinal cord gray matter inflammation to multiple sclerosis pathogenesis. Our findings suggest that activated macrophages/microglia of gray matter are less susceptible to cell death induction. Alternatively, it can be assumed that intrinsic cell death-inductive mechanisms of nervous tissue differ in white and gray matter. Thus, further research on the gray matter macrophages/microglia cell death during EAE is warranted. They should be aimed at identification of the reasons for the observed differences and finding suitable ways to stimulate gray matter activated macrophages/microglia death.

## Introduction

Multiple sclerosis is a chronic, inflammatory, demyelinating and neurodegenerative disease of the central nervous system (CNS). Autoimmune reactivity against CNS antigens is considered a major factor in disease pathogenesis (Sospedra & Martin, 2005). While T helper cells have the dominant role in the initiation of the inflammatory reaction within the CNS (Petermann & Korn, 2011), macrophages are among the major effector cells acting against the CNS (Bogie, Stinissen & Hendriks, 2014). Besides monocytes infiltrating the CNS from the blood, the resident macrophages, including perivascular and meningeal macrophages and microglia, also contribute to CNS tissue destruction (Goldmann & Prinz, 2013; Bogie, Stinissen & Hendriks, 2014). On the other hand, local macrophages are important for limitation of the autoimmune reactivity and for CNS tissue repair (Goldmann & Prinz, 2013; Bogie, Stinissen & Hendriks, 2014).

Cell death of CNS cells, as well as of immune cells infiltrating the CNS, is a basic feature of multiple sclerosis pathogenesis. Both oligodendrocyte and neuronal cell death are considered essential for the disease initiation and progression (Herz, Zipp & Siffrin, 2010; Patel & Balabanov, 2012). Conversely, cell death of inflammatory cells seems to be crucial for the resolution and limitation of the autoimmune response (Pender & Rist, 2001). Nevertheless, there are still unresolved issues regarding cell death within the inflamed CNS, including regional specificity in the susceptibility of immune cells to cell death. The latter is of particular interest as an increasing body of evidence points to the importance of the spinal cord gray matter inflammation and atrophy for the pathogenesis of multiple sclerosis. Gray matter lesions are readily observed by magnetic resonance imaging in post-mortem specimens of the spinal cord obtained from multiple sclerosis patients (Gilmore et al., 2009). It has been recently suggested that the spinal cord gray matter pathology contributes significantly to physical disability in relapsing and secondary progressive multiple sclerosis (Kearney et al., 2014). Moreover, novel data clearly show that spinal cord gray matter atrophy is more pronounced in progressive multiple sclerosis than in relapsing forms of the disease and that it contributes to patient disability more than brain gray matter atrophy (Schlaeger et al., 2014).

Our investigation was focused on cell death detection in the spinal cord of Dark Agouti (DA) rats immunized to develop experimental autoimmune encephalomyelitis (EAE), an animal model of multiple sclerosis. DA rats were our choice as numerous spinal cord gray matter infiltrates are observed in these animals during EAE progression. To elucidate the role of macrophages/activated microglia in the disease phenotype, cell death of ED1^+^ cells was investigated. ED1 is a marker for macrophages also expressed in activated microglia cells, but not in resting microglia (Dijkstra et al., 1985; Milligan, Cunningham & Levitt, 1991; Bauer et al., 1994). Thus, elevated expression of ED1 is an important sign of CNS inflammation. Here, our aim was to detect cell death of these cells at the peak of EAE in DA rats. Dying ED1^+^ cells were detected in large number among infiltrates, as well as in non-infiltrated regions of the spinal cord. The proportion of ED1^+^ cells undergoing cell death was higher in white matter than in gray matter of the spinal cord. Also, it was higher in infiltrated regions of white matter in comparison to non-infiltrated regions of white matter, as well as to infiltrated regions of gray matter. This may imply that inflammatory macrophages/microglia are more efficiently recruited when cell death occurs in white matter than in gray matter.

## Materials and Methods

### Experimental animals and EAE induction

Dark Agouti (DA) rats (female, 2–4 months old) were used in this study. The rats were maintained in the animal facility of the Institute for Biological Research “Siniša Stanković”. Animal manipulation and experimental procedures were approved by the local Ethics Committee (Institute for Biological Research “Siniša Stanković”, No 02-04/15). EAE was induced with rat spinal cord homogenate (SCH) in phosphate buffer saline (PBS, 50% w/v) mixed with equal volume of complete Freund’s adjuvant (CFA, Difco, Detroit, MI) and supplemented with heat-killed *M. tuberculosis* (to 5 mg/ml). Animals were injected with 100 µl of the emulsion intradermally in a footpad of one hind foot. Non-immunized DA rats, age and sex matched were used as control animals. Animals were monitored daily for EAE clinical signs, and scored according to the following scale: 0, no clinical signs; 1, flaccid tail; 2, hind limb paresis; 3, hind limb paralysis; 4, moribund state or death.

### Tissue preparation

Animals from the EAE group were sacrificed at 12–14 days post immunization—d.p.i., corresponding to the highest disability score in the acute phase of the disease. Sex and age-matched naïve animals were used as control. The lumbar regions of spinal cords, where the most numerous lesions and the most intensive inflammation were observed in DA rats (Mattner et al., 2005; Steinbrecher et al., 2005), were used for all further tissue processing. Spinal cords were rapidly dissected on ice. Isolated tissues were fixed in 4% paraformaldehyde solution in 0.1 M phosphate buffer, pH 7.4 for 12 h at 4 °C. For cryoprotection, lumbar regions of spinal cord tissue were transferred into graded sucrose solutions (10, 20, and 30%). The spinal cords were frozen in 2-methyl butane and kept at −80 °C until sectioning. A series of 20 µm thick coronal sections of the lumbar spinal cord (L1–L5) were cut and mounted on Superfrost glass slides, dried for 2 h at room temperature and stored at −20 °C until staining.

### Immunofluorescence and TUNEL assay

Lumbar region of the spinal cords was used for immunofluorescence studies. For immunofluorescence, sections were incubated overnight at 4 °C with primary rabbit antibody against cleaved (activated) caspase 3 (Asp175; 1:300; Cell signalling, Danvers, MA, USA) or primary mouse anti-ED1 antibody (1:200, Abcam, Cambridge, MA, USA) or primary rabbit anti-Iba1 antibody (1:200, Wako, Richmond, VA, USA), then washed in PBS and incubated for 1h with appropriate Alexa Fluor 488- or Alexa Fluor 566-conjugated secondary antibodies (Molecular Probes, Inc. Eugene, USA). Negative controls were carried out by omitting the primary antibody. Sections were mounted with Vectashield with DAPI (Vectorlabs, Burlingame, CA, USA).

Terminal deoxynucleotidyl transferase dUTP nick end labeling (TUNEL) assay was performed using an *in situ* cell death detection kit (Fluorescein or TMR; Roche Diagnostics GmbH, Mannheim, Germany).

### Microscopy, cell counting and statistical analysis

Fluorescence microscopy was performed at room temperature on an Axio Imager Z.1 ApoTome Microscope, equipped with a Zeiss Axiocam MRm digital camera. Images were captured using Zeiss Axiovision 4.7 software; representative pictures were taken from different areas of the spinal cord using a Z stack mode with 20×/0,8 Zeiss Plan-APOCHROMAT objective. Adobe Photoshop CS3 (Adobe Systems Incorporated, San Jose, CA) was used for primary image processing. For quantifications, pictures were captured on at least three transversal sections from five different animals. Six different areas (6,000 µm^2^) were analyzed from every picture. The total number of cells was counted manually as number of DAPI-stained nuclei. The number of positively labeled cells in the spinal cord was counted manually, as well. Values obtained are given as fraction of total cell number in the examined area (i.e., as percentage). Results are presented as mean ± standard error of the mean (SEM) of values obtained from the analyzed areas. For statistical comparisons the unpaired two-tailed Student t-test was employed. *p* < 0.05 was considered statistically significant.

## Results

### Immunization leads to prominent infiltration of both white matter and gray matter of the spinal cord

**Figure 1:**
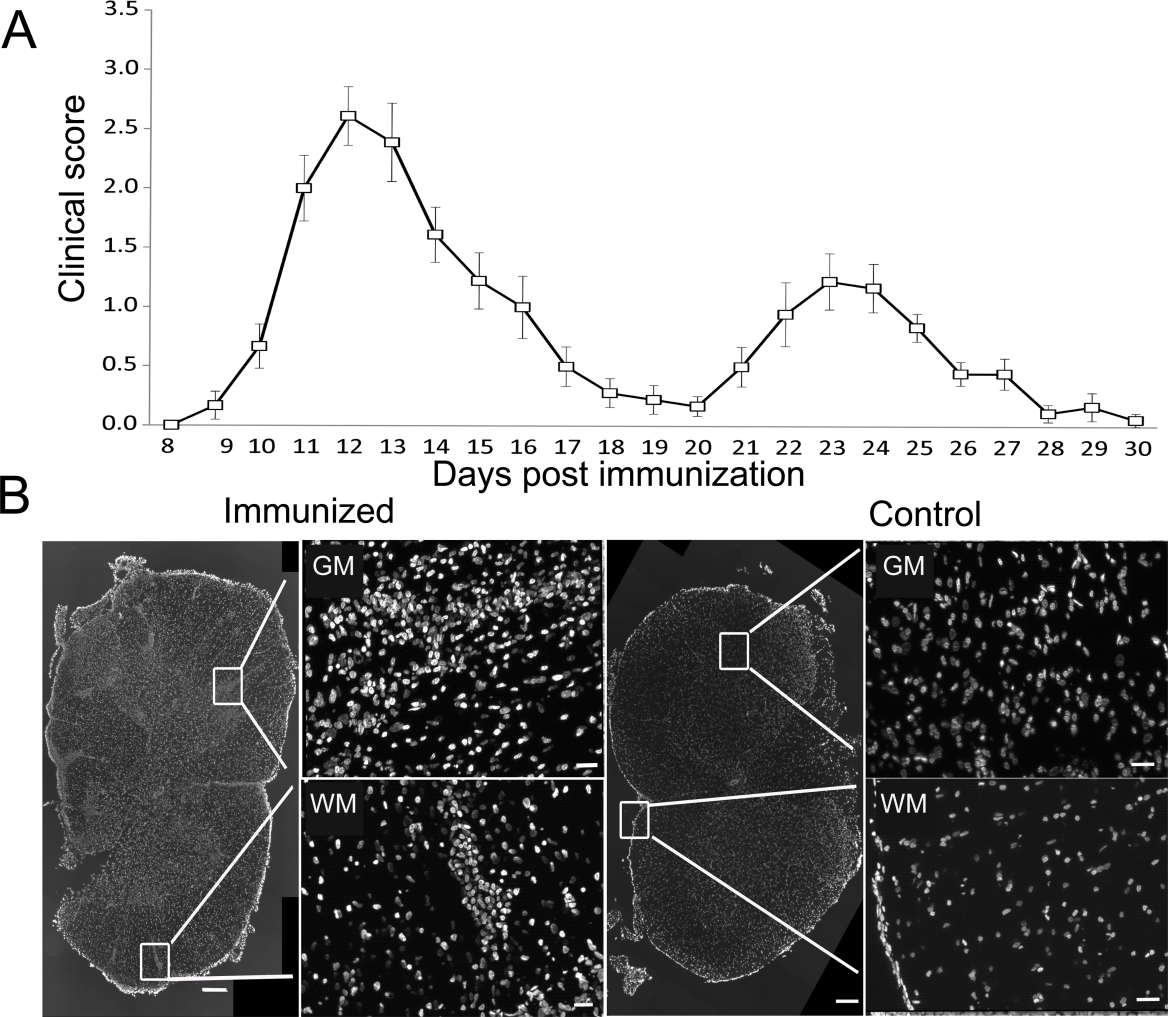
Experimental autoimmune encephalomyelitis in Dark Agouti rats. DA rats were immunized with SCH + CFA, while controls were non-immunized animals. (A) The time course of the neurological disability score was determined on a daily basis and data are presented as mean clinical score + SEM (*n* = 9). (B) Representative sections of spinal cords from immunized and control DA rats stained with DAPI as a nuclear marker. Severe inflammatory infiltrates were present in white and gray matter of immunized animals, as defined by the presence of cellular infiltrates stained with DAPI. WM, white matter; GM, gray matter. Scale bars are 200 and 20 µm for lower and higher magnification pictures, respectively.

DA rats immunized with SCH + CFA showed first clinical signs of EAE on 8–10 days post immunization, while the disease peak characterized by hind limb paralysis was observed on 12–14 days post immunization. To maximize the chances for a positive detection of cell death markers in immune cells, we focused the analysis on the peak of clinical score when a high number of infiltrated immune cells was to be expected (Fig. 1A). Indeed numerous, confluent foci of inflammation were visible in white and gray matter of the spinal cords at the peak of EAE (Fig. 1B). At the same time, spinal cord sections obtained from non-immunized (control) animals showed no signs of infiltration and are presented for comparison (Fig. 1B).

### Numerous ED1^+^ cells are present in infiltrated and non-infiltrated regions of the spinal cords

**Figure 2:**
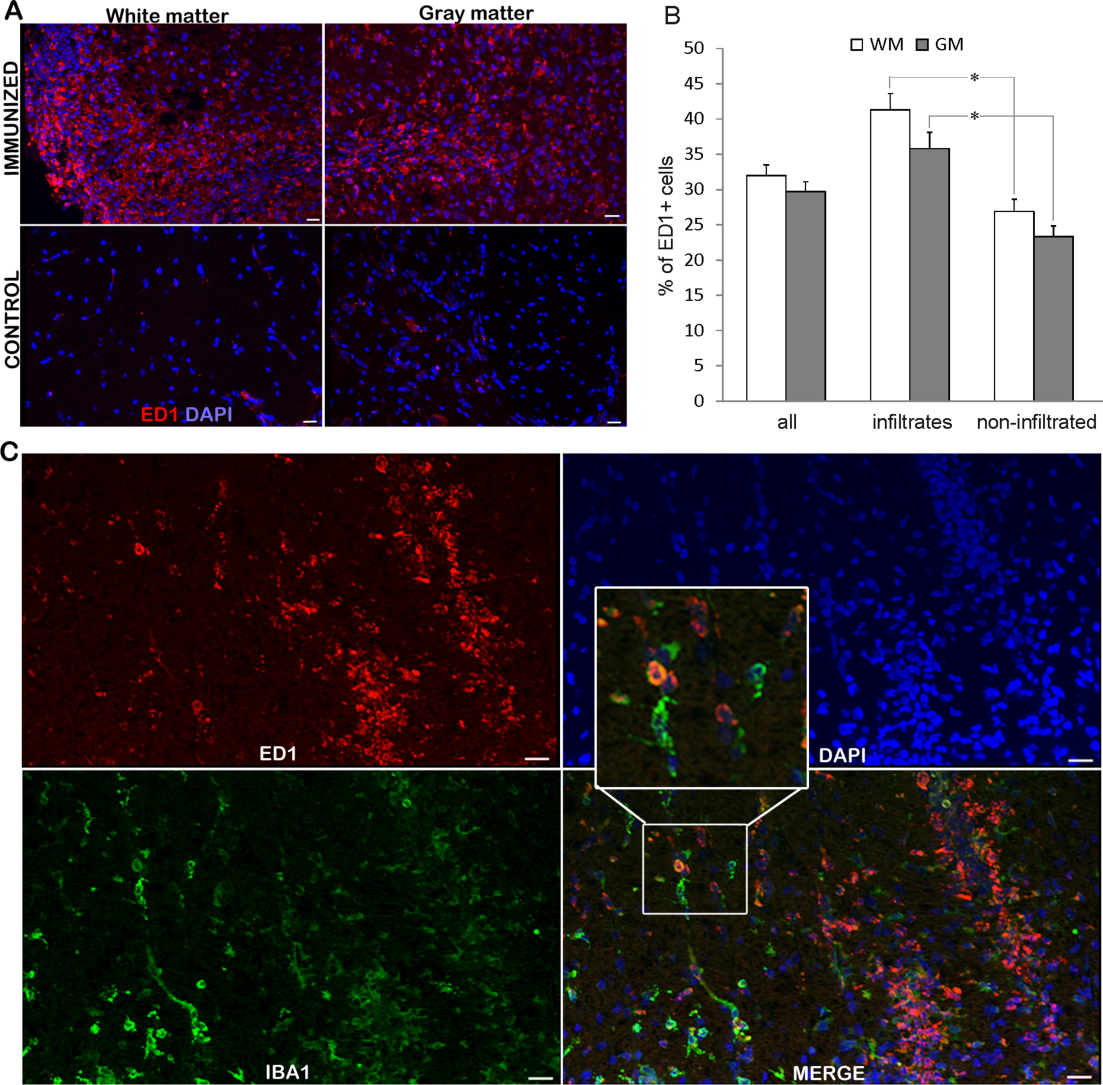
**ED1**^+^ cells invasion into the spinal cords of immunized animals. (A) Prominent inflammatory infiltrates were detected in white and gray matter in immunized animals, where numerous ED1^+^ cells (green) were present infiltrates and non-infiltrated regions, as well. Control sections did not display noticeable ED1 immunoreactivity. (B) The percentage of ED1^+^ cells among white matter and gray matter cells in whole spinal cord (all) or within infiltrates or in non-infiltrated regions is presented as mean + SEM. (C) ED1^+^ cells (red) and Iba1^+^ cells (green) were present in infiltrated and non-infiltrated regions. Both single-positive and double-positive cells were observed. WM, white matter; GM, gray matter. *p < 0.05 represents statistically significant differences between infiltrates and non-infiltrated regions. Scale bars are 20 µm.

While ED1^+^ cells were hardly detectable in the spinal cords of non-immunized rats, numerous positive cells were visible in the spinal cords at the peak of EAE (Fig. 2A). ED1^+^ cells were present in white and gray matter, both among infiltrating cells and in non-infiltrated regions. The observed high percentage of ED1^+^ cells, around 40% (Table 1 and bar graph in Fig. 2B), in the region of high infiltration is in accordance with our previous observation of the cellular composition of the infiltrates (Miljković et al., 2011). At the same time, a relatively high percentage of ED1^+^ cells (around 25%) was observed in non-infiltrated regions. Nevertheless, the number of ED1^+^ cells was significantly higher in infiltrated than in non-infiltrated regions. No difference was detected between white matter and gray matter irrespectively if infiltrated or non-infiltrated regions were compared. Spinal cord sections were also stained for Iba1 and it was shown that neither all Iba1 cells in non-infiltrated regions were positive for ED1^+^, nor all ED1^+^ cells within infiltrates were Iba1 positive (Fig. 2C). Thus, activated macrophages/microglia were present throughout the spinal cord, possibly as a consequence of local activation of microglia, as well as of infiltration of macrophages.

**Table 1:** ED1^+^ cells in the spinal cord.

		ED1^+^ (%)
Complete spinal cord	GM and WM	31.0 ± 1.0
	WM	32.0 ± 1.5
	GM	29.7 ± 1.4
Infiltrated region	GM and WM	39.2 ± 1.7
	WM	41.3 ± 2.3
	GM	35.8 ± 2.3
Non-infiltrated region	GM and WM	25.5 ± 1.2^*^
	WM	26.9 ± 1.7^*^
	GM	23.3 ± 1.5^*^

**Notes.**

WM white matter GM gray matter

* *p* < 0.05 infiltrated vs. non-infiltrated region.

### Apoptotic and non-apoptotic cell death in spinal cord

**Figure 3:**
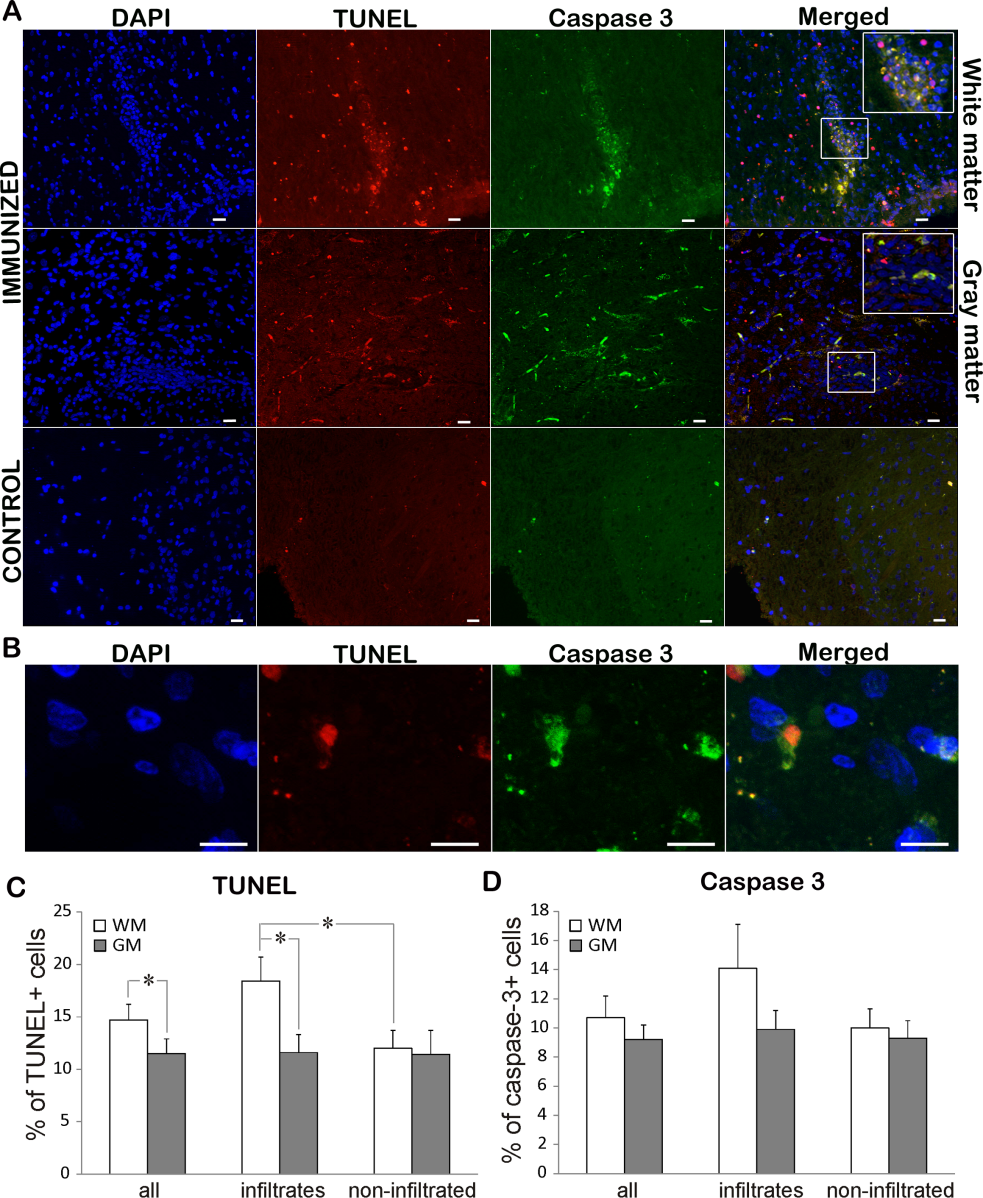
Cell death in EAE rats at the disease peak. (A) Representative spinal cord sections of immunized and control animals stained for nuclei with DAPI (blue), cleaved caspase 3 (green) and cell death with TUNEL (red) are presented. TUNEL cells were present both in infiltrates and non-infiltrated regions in white matter, while caspase 3 positive cells were more prominent in infiltrates. The merged pictures showed that vast majority of caspase 3 positive cells were TUNEL positive, while there was a population of TUNEL cells which were caspase 3 negative. Inserts in merged pictures are shown at higher magnification in the upper right corner. (B) Higher magnification of cleaved caspase 3 and TUNEL co-labeling, where caspase 3 staining (green) is visible in cytoplasm, while TUNEL staining (shown in red) is confined to nucleus. (C) The percentage of TUNEL and (D) caspase 3 positive cells within white matter and gray matter cells throughout spinal cord (all) or within infiltrates or in non-infiltrated regions is presented as mean + SEM. WM, white matter; GM, gray matter. *p < 0.05 represents statistically significant difference between infiltrates vs. non-infiltrated regions or white matter vs. gray matter. Scale bars are 20 µm.

To assess the rate of cell death in EAE rats, TUNEL staining was performed on both immunized and control animals. While TUNEL positive cells were rarely detected in control sections, immunization led to extensive cell death in both white and gray matter (Figs. 3A, 3C; Table 2). There were significantly more TUNEL cells in white matter than in gray matter. At the same time there were more dying cells in infiltrated vs. non-infiltrated regions in the white matter. On the other hand, there was no difference in the percentage of dying cells in infiltrated and non-infiltrated regions within the gray matter (11.6 ± 1.7 vs. 11.4 ± 2.3). To determine if the observed cell death followed the “classical” apoptotic pathway characterized by caspase 3 activation, we looked for cleaved and activated caspase 3 positive cells (Figs. 3A and 3D). Caspase 3 positive cells essentially followed the pattern of TUNEL cells, with majority of caspase 3 positive cell also being positive for TUNEL (Fig. 3A). In most of the analyzed cells, caspase 3 staining was observed in the cytoplasm, while the TUNEL assay marked the nucleus (Fig. 3B). In some cells, the TUNEL signal traced the morphology of the cell and was morphologically co-labeled with cleaved caspase-3, as can be seen in Fig. 3A. This can be explained by the known fact that TUNEL labels DNA nick-ends, which in very late stages of cell death after the nucleus has disintegrated can also be found in the cytoplasm (Labat-Moleur et al., 1998). Of special interest is the observation that only about 70% of TUNEL positive cells were caspase 3 positive (Table 2), suggesting that a certain cell population was dying using a caspase 3-independent pathway.

**Table 2:** Cell death in the spinal cord.

		TUNEL^+^ (%)	Caspase 3^+^ (%)	Caspase 3^+^ in TUNEL^+^ (%)
Complete spinal cord	GM and WM	13.2 ± 1.0	10.9 ± 1.0	71.5 ± 4.8
	WM	14.7 ± 1.3	10.7 ± 1.4	70.9 ± 5.0
	GM	11.5 ± 1.4^**^	9.2 ± 1.0	73.6 ± 10.0
Infiltrated region	GM and WM	14.7 ± 1.4	12.9 ± 1.7	75.8 ± 6.3
	WM	18.4 ± 2.2	14.1 ± 3.0	75.8 ± 7.0
	GM	11.6 ± 1.7^**^	9.5 ± 1.4	75.7 ± 9.8
Non-infiltrated region	GM and WM	11.7 ± 1.3	9.8 ± 1.0	67.0 ± 6.7
	WM	12.0 ± 1.5^*^	10.0 ± 1.4	65.9 ± 7.6
	GM	11.4 ± 2.3	9.3 ± 1.2	71.4 ± 10.1

**Notes.**

WM white matter GM gray matter

* *p* < 0.05 infiltrated vs. non-infiltrated region.

** *p* < 0.05 WM vs. GM.

### Dying ED1^+^ cells are prevalent in white matter

**Figure 4:**
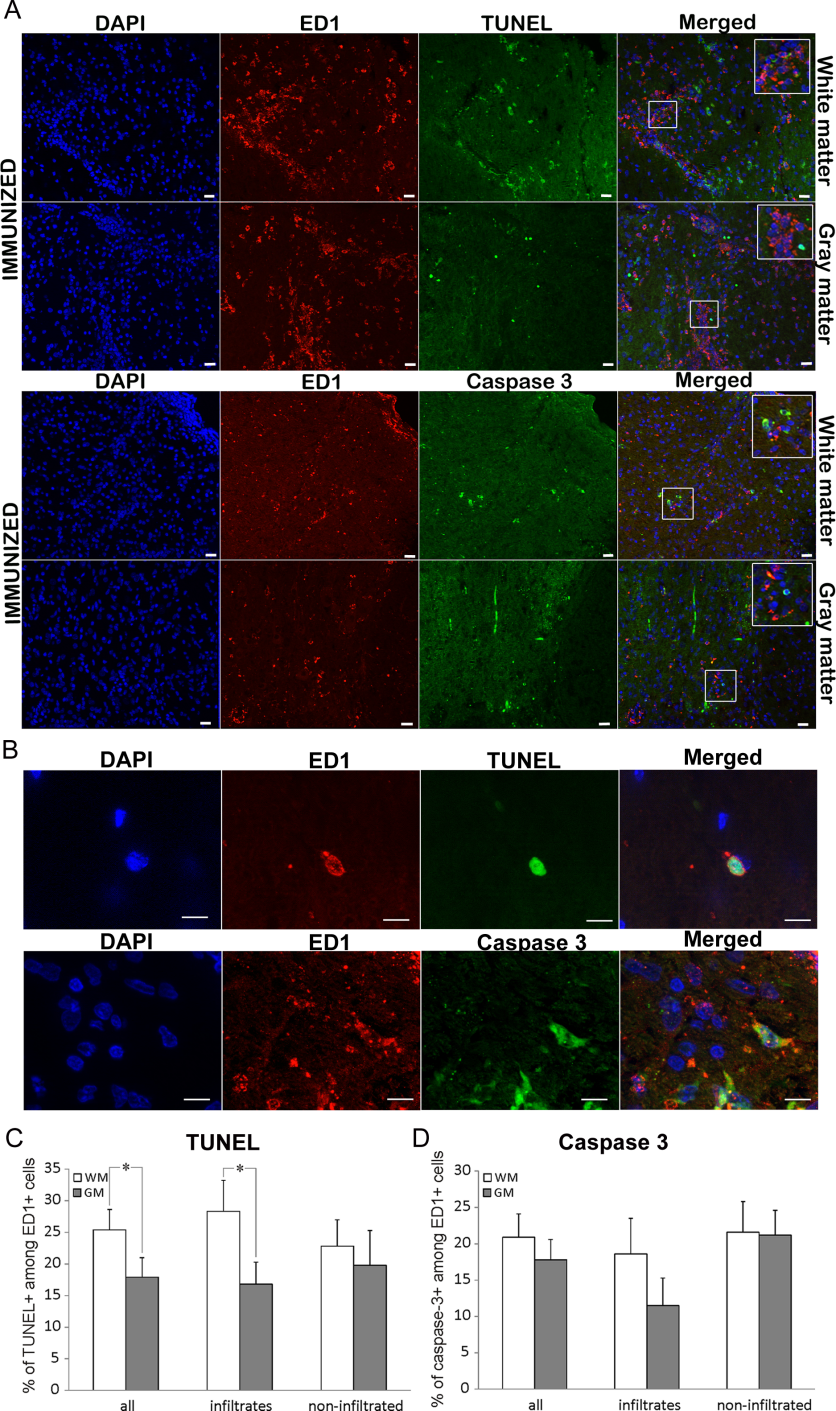
Cell death of **ED1**^+^ cells in immunized animals (A) Spinal cord cross-sections of immunized animals were stained for ED1^+^ cells (red) and for dying cells (green). There were more dying cells among ED1^+^ cells in white matter compared to gray matter. (B) White and gray matter from immunized rats were also co-stained for ED1^+^ (red) and cleaved caspase 3 (green) cells. The merged pictures showed cells positive for each of the markers as well as cells positive for both stainings. Inserts represent a higher magnification from infiltrated regions of both white matter and gray matter. (C) Bar graphs represent the percentages of TUNEL positive cells among ED1^+^ cells in white matter (WM) and gray matter (GM), in whole spinal cord (all), or within infiltrates, or in non-infiltrated regions. (D) The percentage of caspase positive among ED1^+^ cells showed no statistically significant difference between white matter and gray matter, or between infiltrates and non-infiltrated regions. Quantitative data are presented as mean + SEM. *p < 0.05 represents statistically significant difference between infiltrates vs. non-infiltrated regions or between white matter. Scale bars are 20 µm.

Co-staining for ED1 and TUNEL was performed in order to attribute the role of ED1^+^ cells in spinal cord cell death as the consequence of the immunization. ED1^+^ cells positive for TUNEL were detected within the infiltrates, as well as in non-infiltrated regions (Figs. 4A–4C). About 40% of all the TUNEL positive cells colocalized with ED1 staining (Table 3). This percentage remained relatively constant both in white matter and gray matter as well as in infiltrates and non-infiltrated regions. However, when the proportion of TUNEL positive cells among ED1^+^ cells was assessed, a significant difference was detected between white matter and gray matter in general (25.4 ± 3.2 and 17.9 ± 3.1, respectively). Still, the significance was confined only to infiltrated regions of white matter and gray matter (Table 3).

**Table 3:** Cell death of **ED1**^+^ cells in the spinal cord.

		ED1^+^ in TUNEL (%)	TUNEL in ED1^+^ (%)	ED1^+^ in casp 3 (%)	Casp 3 in ED1^+^ (%)
Complete spinal cord	GM and WM	44.0 ± 3.5	22.3 ± 2.3	30.7 ± 3.7	20.2 ± 2.2
	WM	43.9 ± 4.8	25.4 ± 3.2	26.1 ± 4.4	20.9 ± 3.1
	GM	44.1 ± 5.3	17.9 ± 3.1^**^	37.3 ± 6.1	17.8 ± 2.8
Infiltrated region	GM and WM	48.1 ± 4.7	22.0 ± 3.0	30.9 ± 6.0	17.7 ± 3.6
	WM	47.5 ± 6.8	28.3 ± 4.9	30.4 ± 7.2	20.1 ± 3.5
	GM	48.7 ± 6.6	16.7 ± 3.5^*^	23.2 ± 6.4	12.5 ± 3.9
Non-infiltrated region	GM and WM	39.2 ± 5.3	22.3 ± 3.4	30.0 ± 4.4	21.5 ± 3.0
	WM	40.8 ± 6.7	23.6 ± 4.3	25.3 ± 5.2	21.7 ± 3.9
	GM	36.5 ± 8.7	20.7 ± 5.7	42.4 ± 8.2	21.7 ± 3.5

**Notes.**

WM white matter GM gray matter

* *p* < 0.05 infiltrated vs. non-infiltrated region.

** *p* < 0.05 WM vs. GM.

### ED1^+^ cells in the spinal cord die in a caspase 3-dependent fashion

To determine if the predominant ED1^+^ cell death mechanism was apoptotic, co-labeling of ED1 and caspase 3 was employed. Cells positive for cleaved caspase 3 were detected among ED1^+^ cells both within the infiltrates, and in non-infiltrated regions of spinal cord (Figs. 4A, 4B and 4D). The proportion of caspase 3 positive cells among ED1^+^ cells was numerically higher in white than in gray matter, as well as in infiltrated regions in white matter in comparison to gray matter infiltrates, but the differences were not statistically significant (Table 3). Although there was a substantial number of ED1^+^ cells among caspase 3 positive population, no differences in the analyzed regions of the spinal cords were detected (Table 3).

## Discussion

Cell death is one of the most important processes in the pathogenesis of multiple sclerosis and EAE. Here, cell death of ED1^+^ cells, i.e., macrophages and activated microglia, in infiltrated and non-infiltrated regions of gray matter and white matter of DA rat spinal cord at the peak of EAE was detected. We show that the proportion of dying ED1^+^ cells, i.e., the macrophages and activated microglia was higher in white matter than in gray matter, particularly when infiltrated regions were compared.

The percentages of ED1^+^ cells determined in the infiltrated regions of the spinal cord are very similar to the proportion of CD4^low^CD11b^+^ that we have previously observed among immune cells isolated from the spinal cord (Miljković et al., 2011). Since the CD4^low^CD11b^+^ phenotype also corresponds to activated macrophages and microglia (Sedgwick et al., 1991), our previous and current data are in strong agreement. Importantly, activated macrophages/microglia were observed in infiltrated and not infiltrated regions of both white and gray matter of the spinal cord. This is consistent with data obtained in human samples, as activation of microglia and/or macrophages was shown in active and inactive multiple sclerosis lesions, but also in non-affected white matter and in cortical gray matter lesions (details in Abourbeh et al., 2012). Proliferation is an important feature of microglial activation and it has been reported that microglial cells proliferate in MS and EAE lesions (Schönrock et al., 1998; Ponomarev et al., 2005). It was reported that more than one third of total macrophage and dendritic cell populations observed in the inflammatory lesions at the peak of EAE were activated microglial cells (Ponomarev et al., 2005). Moreover, these cells co-localized with infiltrating leukocytes in the lesions (Ponomarev et al., 2005). Thus, the cells present in the regions of the spinal cord that are defined as infiltrated regions in our study are mixture of blood-borne immune cells and proliferating microglia. Similar conclusions can be drawn from the simultaneous staining of spinal cord sections for Iba1 and ED1. Iba1 is considered as a general marker for microglial cells (Ito et al., 1998), yet its expression cannot be correlated with microglial activation (Xue et al., 2010). On the other hand, ED1 is a marker of altered cellular activity of microglia, as it indicates phagocytic activity of these cells (Bauer et al., 1994). Still, not all microglial cells express this molecule (Xue et al., 2010). Importantly, not all ED1^+^ cells in infiltrates were Iba1 positive, thus showing that besides microglial cells, macrophages were also present in white and gray matter infiltrates. Furthermore, not all Iba1 positive cells in non-infiltrated regions were ED1^+^, hence indicating that not all of microglial cell were phagocytically competent at the peak of EAE.

The proportion of apoptotic cells detected in the spinal cord of DA rats in our study is similar to that observed in Lewis rats at the peak of EAE (White, McCombe & Pender, 1998). Apoptosis in Lewis rats was detected by cell cycle analysis as a fraction of hypodiploid cells among immune cells isolated from the spinal cord. Apoptotic cells were detected both in microglia and macrophages and the percentage of dead cells was again similar to the proportion of dying ED1^+^ cells observed in our study. In our study, majority of caspase 3 positive cells were also positive for the TUNEL assay. Still, there were around 30% of cells in the spinal cord of DA rats that were not dying in a caspase 3-dependent way. Since similar percentages of caspase 3 and TUNEL positive cells were observed among ED1^+^ cells, it is plausible to assume that the majority, if not all, of the ED1^+^ cells died in a caspase 3-dependent way, in the spinal cord of DA rats, at the peak of EAE. This assumption is in accordance with previous reports on caspase 3-dependent apoptosis as the way for macrophages/microglia to die in EAE (Nguyen, McCombe & Pender, 1994; White, McCombe & Pender, 1998). Therefore, our data suggest that some other cells, negative for ED1 were dying in a non-apoptotic way. Previously it was suggested that target and not effector cells die in a non-apoptotic way in EAE (Bonetti et al., 1997). For instance, it was shown that oligodendrocytes die in a caspase 3-independent way in multiple sclerosis (Barnett & Prineas, 2004). In addition, we have previously shown that a single neuronal cell is able to execute apoptotic and non-apoptotic cell death pathways, depending on the exact physiological status of the cell (Arango-Gonzalez et al., 2014; Trifunović et al., 2012). The further identification of cells dying in non-apoptotic ways and the precise dissection of the molecular mechanisms involved in cell death regulation in these cells in the DA rat CNS is the aim of our ongoing study.

Significantly less dying ED1^+^ cells were observed in gray matter than in white matter of EAE rats. Gray matter is becoming increasingly appreciated as the major area of the pathology in multiple sclerosis, even in the earliest phases of the disease (Calabrese et al., 2015). Gray matter lesions are observed in the spinal cord of multiple sclerosis patients (Gilmore et al., 2009) and they are suggested to contribute significantly to physical disability in relapsing and secondary progressive multiple sclerosis (Kearney et al., 2014). Spinal cord gray matter atrophy seems to be particularly important for the pathogenesis of progressive multiple sclerosis (Schlaeger et al., 2014). The fact that spinal cord gray matter pathology is a characteristic of DA rat EAE highlights the importance of this model for multiple sclerosis research. A lower incidence of cell death among ED1^+^ cells in spinal cord gray matter in comparison to white matter in DA rat EAE implies that the activated macrophages/microglia of gray matter are less susceptible to cell death induction. This resistance of gray matter macrophages/microglia might come as a consequence of specificity in intrinsic cell death-inductive mechanisms of gray matter nervous tissue in comparison to white matter nervous tissue. Also, it is possible that the infiltrating cells in these two regions are at different pathological stages. Therefore, more detailed kinetic studies on the cell death of activated macrophages/microglia in EAE are warranted.

Since macrophages/microglia have both detrimental and beneficial roles in multiple sclerosis and EAE, less ED1^+^ cell death in gray matter could have divergent significance for the CNS pathology. Inefficient induction of activated macrophages/microglia cell death in gray matter might contribute to neuronal and axonal injury. Indeed, it has been elaborated that activated macrophages/microglia produce and release various cytotoxic molecules, including reactive oxygen and nitrogen species (Lassmann, 2010). Along the same line, axonal injury in multiple sclerosis has been associated with the presence of activated macrophages/microglia in the vicinity of axons (Lassmann, 2010). On the contrary, less cell death of alternatively activated macrophages/microglia might improve the CNS pathogenesis. Alternatively, activated macrophages/microglia produce anti-inflammatory cytokines and other mediators which contribute to the resolution of CNS inflammation and promote nervous tissue repair (Jiang, Jiang & Zhang, 2014). Importantly, it has been recently shown that blood-borne macrophages promote EAE, while microglia-derived macrophages clear debris and suppress cellular metabolism at the disease onset (Yamasaki et al., 2014). This implies that discrepancy in cell death of classical macrophages and microglia-derived macrophages might also contribute to the CNS inflammation in different ways. Thus, future studies should pay a special attention on detailed dissection of macrophage/microglia death in spinal cord gray matter of multiple sclerosis patients.

In conclusion, we show here that there were more dying activated macrophages and microglia in white matter than in gray matter of the spinal cord of rats at the peak of EAE. Having in mind the importance of the spinal cord gray matter inflammation for the pathogenesis of EAE and multiple sclerosis, further studies on the cell death of ED1^+^ cells in DA rats are warranted. They should aim at a detailed temporal investigation of ED1^+^ cell death during the course of EAE. Also, additional markers of macrophages/microglia, including Iba1 and F4/80 may be analyzed in parallel. Finally, a modulation of ED1^+^ cell death within the CNS should be explored as a therapeutic approach for the treatment of neuroinflammation in multiple sclerosis.

## Supplemental Information

10.7717/peerj.1189/supp-1 Data S1 Raw data
